# Attenuation of the Hepatoprotective Effects of Ileal Apical Sodium Dependent Bile Acid Transporter (ASBT) Inhibition in Choline-Deficient L-Amino Acid-Defined (CDAA) Diet-Fed Mice

**DOI:** 10.3389/fmed.2020.00060

**Published:** 2020-02-25

**Authors:** Anuradha Rao, Ivo P. van de Peppel, Sanjeev Gumber, Saul J. Karpen, Paul A. Dawson

**Affiliations:** ^1^Department of Pediatrics, Emory University School of Medicine, Atlanta, GA, United States; ^2^Section of Molecular Metabolism and Nutrition, Department of Pediatrics, University Medical Center Groningen, University of Groningen, Groningen, Netherlands; ^3^Division of Pathology, Yerkes National Primate Research Center, Emory University, Atlanta, GA, United States

**Keywords:** liver, triglyceride, fibrosis, fat absorption, cholesterol

## Abstract

Non-alcoholic fatty liver disease (NAFLD) is a major growing worldwide health problem. We previously reported that interruption of the enterohepatic circulation of bile acids using a non-absorbable apical sodium-dependent bile acid transporter inhibitor (ASBTi; SC-435) reduced the development of NAFLD in high fat diet fed mice. However, the ability of ASBTi treatment to impact the progression of NAFLD to non-alcoholic steatohepatitis (NASH) and fibrosis in a diet-induced mouse model remains untested. In the current study, we assessed whether ASBTi treatment is hepatoprotective in the choline-deficient, L-amino acid-defined (CDAA) diet model of NASH-induced fibrosis.

**Methods:** Male C57Bl/6 mice were fed with: (A) choline-sufficient L-amino acid-defined diet (CSAA) (31 kcal% fat), (B) CSAA diet plus ASBTi (SC-435; 60 ppm), (C) CDAA diet, or (D) CDAA diet plus ASBTi. Body weight and food intake were monitored. After 22 weeks on diet, liver histology, cholesterol and triglyceride levels, and gene expression were measured. Fecal bile acid and fat excretion were measured, and intestinal fat absorption was determined using the sucrose polybehenate method.

**Results:** ASBTi treatment reduced bodyweight gain in mice fed either the CSAA or CDAA diet, and prevented the increase in liver to body weight ratio observed in CDAA-fed mice. ASBTi significantly reduced hepatic total cholesterol levels in both CSAA and CDAA-fed mice. ASBTi-associated significant reductions in hepatic triglyceride levels and histological scoring for NAFLD activity were observed in CSAA but not CDAA-fed mice. These changes correlated with measurements of intestinal fat absorption, which was significantly reduced in ASBTi-treated mice fed the CSAA (85 vs. 94%, *P* < 0.001) but not CDAA diet (93 vs. 93%). As scored by Ishak staging of Sirius red stained liver sections, no hepatic fibrosis was evident in the CSAA diet mice. The CDAA diet-fed mice developed hepatic fibrosis, which was increased by the ASBTi.

**Conclusions:** ASBT inhibition reduced intestinal fat absorption, bodyweight gain and hepatic steatosis in CSAA diet-fed mice. The effects of the ASBTi on steatosis and fat absorption were attenuated in the context of dietary choline-deficiency. Inhibition of intestinal absorption of fatty acids may be involved in the therapeutic effects of ASBTi treatment.

## Introduction

Parallel to the global rise in obesity, the disease burden related to non-alcoholic fatty liver disease (NAFLD) is emerging as a major worldwide problem. The number of adults and children with NAFLD is steadily increasing and the current global prevalence is estimated at 24% (1, 2). NAFLD comprises a spectrum of disease states, from non-symptomatic hepatic steatosis to non-alcoholic steatohepatitis (NASH) and liver fibrosis. NAFLD also increases the morbidity and mortality associated with type 2 diabetes mellitus, cardiovascular disease, and chronic kidney disease (3). Unfortunately, an incomplete understanding and lack of experimental NAFLD/NASH animal models that faithfully reproduce the human pathophysiology has slowed the development of new therapies (4). Recently, bile acid-related pathways have emerged as an important therapeutic target for disorders of glucose and lipid metabolism (5). Under physiological conditions, bile acid synthesis and enterohepatic cycling are tightly regulated to maintain a relatively constant whole-body bile acid pool size and restrict the systemic distribution of bile acids. After their secretion along with bile into the duodenum, about 95% of the bile acids are reabsorbed by the apical sodium-dependent bile acid transporter (ASBT; also called the ileal bile acid transporter, IBAT) in the distal small intestine, thereby limiting their flux into the colon. This system is tightly controlled in part by bile acid signaling via the farnesoid-X receptor (FXR) in the liver and intestine. The intestinal microbiota harbor enzymes to deconjugate and convert primary bile acids into secondary bile acids, changing the bile acid pool size, composition, and physicochemical properties (6). These changes alter signaling through FXR and other bile acid-activated receptors, and modulates the metabolic response to bile acids (7).

Several bile acid-based approaches are currently under investigation as potential therapies for liver diseases, including NAFLD. Obeticholic acid, a derivative of the naturally occurring FXR agonist chenodeoxycholic acid (CDCA), improved liver biochemistry and histology scores in NASH patients (8, 9). In addition, we have shown that interruption of the bile acid enterohepatic circulation by an ASBT inhibitor (ASBTi), SC-435, prevented hepatic lipid accumulation and improved markers of NAFLD in mice fed a high fat diet (10). Although these results and similar findings in high fat diet-fed Ldlr^−/−^. Leiden mice demonstrated a robust effect of ASBT inhibition on hepatic lipid accumulation, the effects of ASBT inhibition on the progression from hepatic steatosis to steatohepatitis and fibrosis remain unclear (10, 11). Feeding a Western-type diet (high fat, sucrose, and cholesterol) to mice typically models aspects of the human condition including successfully inducing hepatic lipid accumulation. However, progression from steatosis to NASH and fibrosis is generally limited and highly variable (12–14). Therefore, other dietary, genetic or toxic interventions, or combinations thereof, are required to induce development of NASH in animal models. One widely used intervention for dietary induction of NASH and subsequent fibrosis is the methionine/choline-deficient (MCD) diet. Depriving mice of dietary choline impairs hepatic secretion of very low density lipoproteins (VLDL) and results in hepatic steatosis, oxidative stress, cell death, and increases in cytokine levels (15). Combined with methionine deficiency, the mice develop extensive inflammation after 2 weeks and significant fibrosis after 6 weeks (12). However, MCD diet-fed mice generally lose considerable bodyweight and show no insulin resistance (or even increased sensitivity), conditions that poorly correlate with development of NASH in humans (16, 17). In this current study we assessed the effects of ASBTi treatment on development of NAFLD and fibrosis using a choline-deficient L-amino acid-defined (CDAA) diet, which has been shown to successfully induce hepatic steatosis and subsequent fibrosis after 22 weeks without bodyweight loss (18, 19).

## Materials and Methods

### Animals

Male C57Bl/6J mice aged 10 weeks were obtained from Jackson Laboratories. Animals were group-housed with 4 mice per ventilated cage (Super Mouse 750 Microisolator System; Lab Products) containing bedding (1/8” Bed-O-Cobbs; Andersons Lab Bedding Products) in the same temperature (22° C) and light/dark cycle (12-h; 7 AM to 7 PM) controlled room of the animal facility to minimize environmental differences. Mice were fed *ad libitum* for 22 weeks with either a choline-sufficient L-amino acid-defined control diet (CSAA, Catalog # 518754; Dyets Inc., Bethlehem, PA, USA) or choline-deficient L-amino acid-defined diet (CDAA, Catalog # 518753; Dyets Inc., Bethlehem, PA, USA) with or without 0.006% (w/w) of the ASBTi (SC-435). This amount of ASBTi in the diet provides a dose of ~11 mg/kg/day of SC-435 (10). The CSAA and CDAA diets contain 31% of calories as fat (in the form of corn oil and partially hydrogenated vegetable oil; fatty acid composition: 23.4% saturated, 52.1% monounsaturated, 24.5% polyunsaturated) and have no added cholesterol. Food consumption was measured by weighing new and remaining food two times weekly. Calorie intake was calculated by multiplying the weight of the food consumed by their caloric density. During the final week on diet, the mice were individually housed to measure fat absorption and fecal bile acid and neutral sterol excretion.

### Animal Experiments

During the final week of CSAA and CDAA (+/− ASBTi) diet feeding, mice received powdered diet containing 0.7% sucrose polybehenate (w/w) (20). Sucrose polybehenate is not absorbed in the intestine. Therefore, by comparing the ratio of behenic acid to total fatty acids in the diet and feces, the fractional fat absorption can be calculated without being reliant on food intake measurements. Powdered diet was placed in a feeding jar in the cage and replaced every 2–3 days. Feces were collected for individual mice during the final 3 days of the experiment and used for fatty acid, neutral sterol and bile acid measurements. After, mice were sacrificed and tissues collected for further analysis. Mice were anesthetized using isoflurane. Blood was obtained via cardiac puncture, centrifuged and the plasma was stored at −80°C. Livers were excised, weighed and pieces collected for subsequent histology analysis. Remaining liver tissue was snap frozen in liquid nitrogen. The small intestines were excised, measured, cut into five equal length segments, flushed with ice cold phosphate buffered solution (PBS) and snap frozen in liquid nitrogen. The length of the colon was measured, divided into proximal (60%) and distal (40%) segments, flushed with ice cold PBS and immediately snap frozen in liquid nitrogen.

### Fatty Acid Measurements

Weighed samples of the synthetic diet and feces were saponified with methanolic NaOH, extracted with hexane, and converted to methyl esters. Samples were analyzed using gas chromatography (GC) to quantitate the fatty acid methyl esters and determine the amount of behenic acid (C22:0), saturated (14:0, 16:0, 18:0), monounsaturated (C18:1), and polyunsaturated (18:1, 18:2, 18:3ω3, 20:5ω3, 22:6ω3) fatty acids as previously described (21, 22). Each chromatogram was examined to verify the identification of constituent fatty acids and for quality control.

### Histology

The livers were removed, weighed, and a portion was fixed in 10% neutral buffered formalin, embedded in paraffin, sectioned at 5 μm, and stained with hematoxylin and eosin. Sirius red staining was performed using the paraffin-embedded liver sections (method adapted from Picrosirius Red Stain Kit, Polysciences, Inc., Warrington, PA, USA). The liver histology was assessed in a blinded fashion by a veterinary pathologist (S.G.) for steatosis, lobular inflammation, and hepatocellular ballooning to derive the NAFLD Activity Score (NAS) as described (23). Sirius red stained sections were blindly assessed by S.G. for Ishak Stage using the scores adapted from Ishak et al. (24).

### Hepatic and Jejunal Lipids

Hepatic lipids were extracted according to a protocol based on the Folch method (25). Briefly, lipids were extracted from ~60 mg of tissue using 3 ml of chloroform:methanol (2:1) and incubated at 55°C for at least 2 h. Phases were split by adding 0.05% (v/v) sulfuric acid in water and centrifugation at 1,500 rpm for 15 min. Part of the bottom layer was transferred, dried under nitrogen and dissolved in 2% (v/v) Triton X-100 in water. Hepatic concentrations of total cholesterol (Pointe Scientific, C7510-01-906), free cholesterol (Fujifilm Wako Diagnostics, Cat# 993-02501), and triglyceride (Fujifilm Wako Diagnostics, Cat# 994-02891 and Cat# 990-02991) were subsequently measured by enzymatic assays.

### Gene Expression

Total RNA was isolated from proximal colon and liver using a miRNeasy kit (Qiagen, Cat# 74106). Reverse transcriptase polymerase chain reaction (RT-PCR) was performed with 1 μg RNA using a high capacity cDNA reverse transcription kit (Applied Biosystems, Cat# 4368814). Real-time quantitative PCR was performed with a Sybr Green master mix (Applied Biosystems, Cat# 4309155) using a StepOne Plus real time PCR system (Applied Biosystems). The mRNA expression levels were calculated based on the ΔΔ-CT method; values are means of triplicate determinations and expression was normalized using cyclophilin. The sequences of the primers used has been published previously (26).

### Fecal Bile Acids and Neutral Sterols

Fecal pellets were sorted, air-dried, weighed and mechanically homogenized. Neutral sterols and bile acids were extracted from 50 mg of feces or diet as described (27). Briefly, samples were heated for 2 h at 80°C with a mixture of 1 M sodium hydroxide and methanol (3:1). Neutral sterols were then extracted twice with 2 ml petroleum ether and derivatized with *N, O*-Bis(trimethylsilyl)trifluoroacetamide (BSTFA)-pyridine-trimethylchlorosilane (TMCS) (5:5:0.1). Bile acids were quantitatively extracted from feces, isolated on Sep-Pak C-18 columns, methylated with methanol/acetyl chloride (20:1) and derivatized with BSTFA-pyridine-TMCS (5:5:0.1) (27). Both neutral sterols and bile acids were measured by gas chromatography (GC) (28). The total amount of bile acids or neutral sterols was calculated as the sum of the individual species.

### Statistical Analyses

Data are presented as means ± standard deviation (SD), unless stated otherwise. Statistical analyses were performed and graphs were created using GraphPad Prism 8 (GraphPad Software, La Jolla, CA, USA). Differences between groups were assessed by one-way ANOVA with Tukey's *post-hoc* test except for Ishak Stage, which was tested using a Chi-Squared test. Different lowercase letters indicate statistically significant differences (*P* < 0.05) between groups.

## Results

### ASBT Inhibitor Treatment Reduces Bodyweight Gain in CSAA and CDAA Diet-Fed Mice

**Figure 1:**
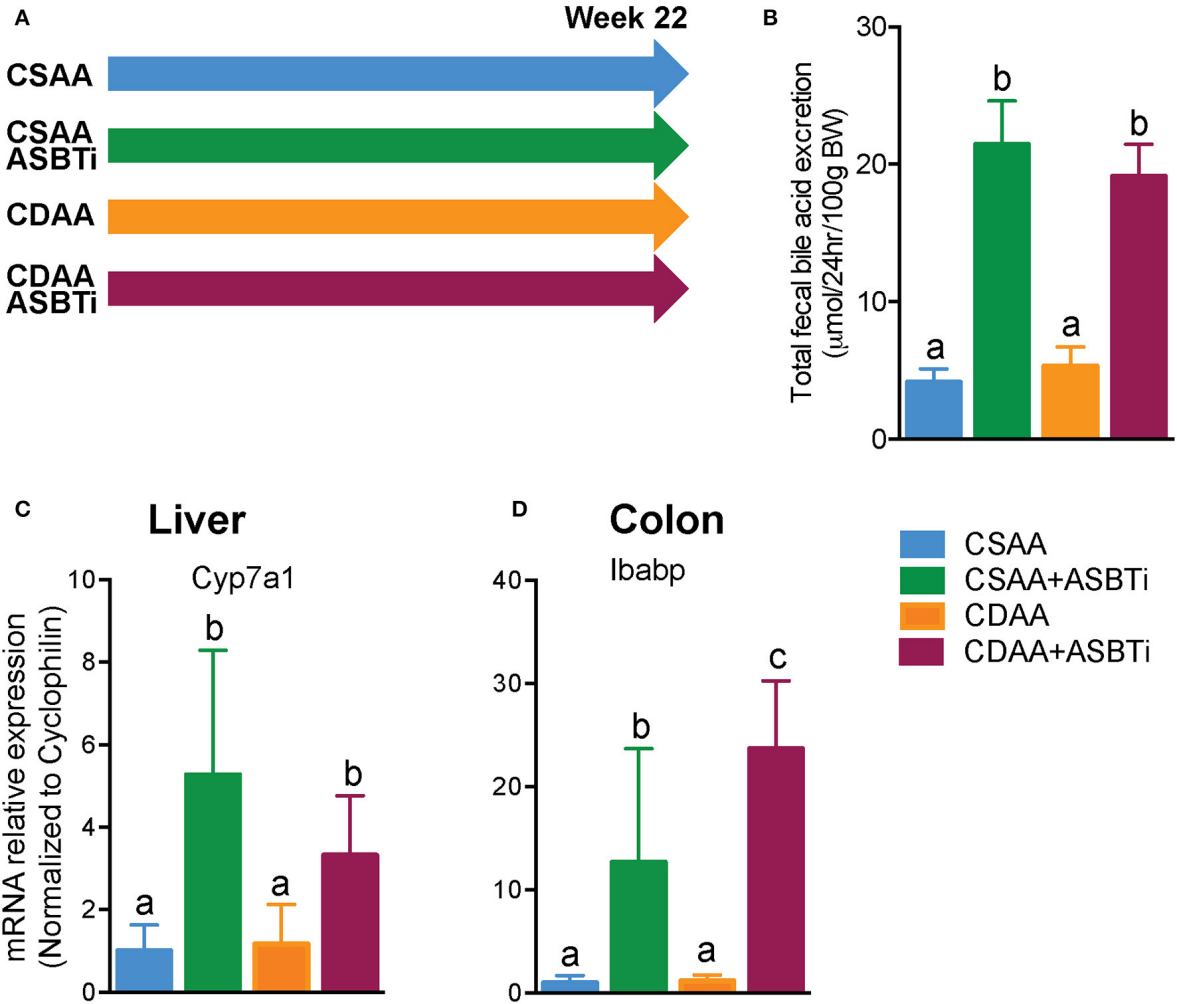
ASBTi treatment increases fecal bile acid excretion and bile acid synthesis in mice fed a CSAA and CDAA diet. **(A)** Study design showing the four experimental groups, **(B)** Total fecal bile acid excretion, **(C)** Hepatic Cyp7a1 gene expression, **(D)** Colonic Ibabp gene expression. Means ± SD are shown. Distinct lowercase letters indicate significant differences between groups. *P* < 0.05; *n* = 9–12 per group.

To determine whether ASBT inhibition can prevent development of NASH and fibrosis in mice, male C57Bl/6J mice were fed a choline-deficient L-amino-defined diet (CDAA) or a choline-sufficient control diet (CSAA) with or without an ASBTi for 22 weeks (Figure 1A). ASBTi treatment increased total fecal bile acid excretion by approximately 5-fold in CSAA (4.1 vs. 21.4 μmol/24 h/100 g BW, *P* < 0.001) and 4-fold in CDAA diet-fed mice (5.3 vs. 19.1 μmol/24 h/100 g BW, *P* < 0.001) (Figure 1B). Interruption of the enterohepatic circulation of bile acids was confirmed by gene expression measurements, with increases in hepatic cholesterol 7alpha-hydroxylase (*Cyp7a1*) mRNA as a marker of bile acid synthesis, and colonic intestinal bile acid-binding protein (*Ibabp*) mRNA as a marker of colon bile acid exposure (Figures 1C,D) in both the CSAA and CDAA diet-fed mice.

**Figure 2:**
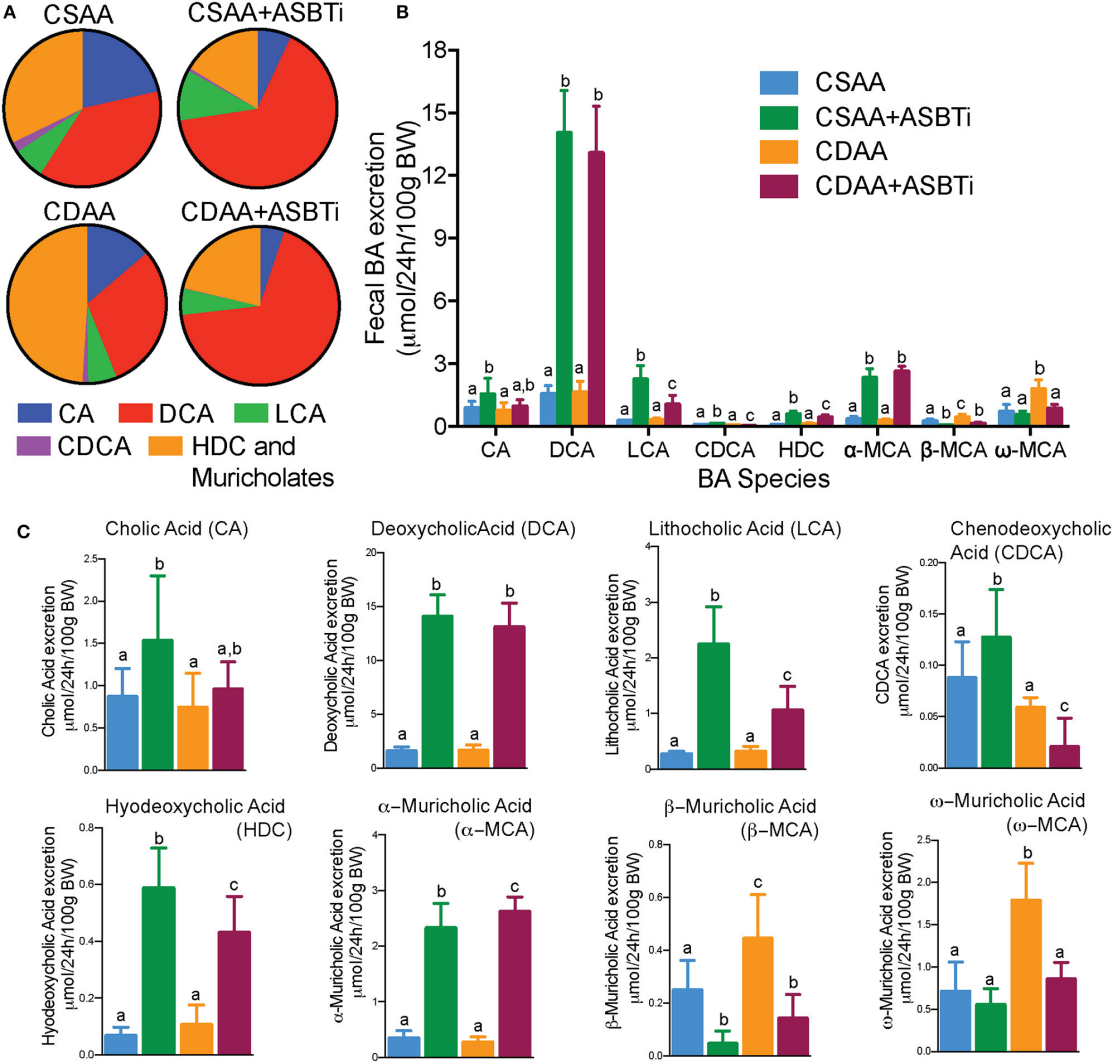
The effects of ASBTi treatment on the fecal bile acid species profile. ASBTi treatment increased excretion of the secondary bile acids, deoxycholic acid (DCA) and lithocholic acid (LCA), and the primary bile acid, α-muricholic acid (MCA). **(A)** Pie charts for the fecal bile acid profiles. **(B)** Mass of bile acid species excreted into the feces per day. **(C)** Individual species of BA excreted in feces. Means ± SD are shown. Distinct lowercase letters indicate significant differences between groups. *P* < 0.05; *n* = 8–12 per group.

Inhibiting the ASBT in mice has been shown to increase the proportion of cholic acid (CA) plus its bacterial dehydroxylation product deoxycholic acid (DCA) and to reduce the proportion of 6-hydroxylated bile acid species, including alpha-muricholic acid (αMCA), beta-muricholic acid (βMCA), and the bacterial product omega-muricholic acid (ωMCA) (10, 11, 29). To determine if the CDAA diet alters the effect of ASBT inhibition on bile acid composition, the fecal bile acid profiles were determined using quantitative three-day fecal collections performed for individually-housed mice (Figure 2). The mass of feces excreted per day per unit body weight (g dry feces/24 h/100 g BW, mean ± SD, *n* = 9–12/group) was similar for the CSAA vs. CDAA diet-fed mice, showed a trend toward an increase in ASBTi-treated CSAA diet-fed mice, and was significantly decreased in ASBTi-treated CDAA diet-fed mice (CSAA: 1.100 ± 0.266 vs. CSAA plus ASBTi: 1.326 ± 0.169, *P* = 0.088; CDAA: 1.157 ± 0.230 vs. CDAA plus ASBTi: 0.908 ± 0.071, *P* = 0.02). The fecal bile acid results are expressed as a proportion of the total amount of bile acid excreted per day per unit body weight (Figure 2A) and as the mass of individual bile acid species excreted per day per unit body weight (Figures 2B,C). For the fecal bile acid profiles, the proportion of βMCA and its bacterial product ωMCA were increased and the proportion of CA and its bacterial product DCA decreased in the CDAA vs. CSAA diet-fed mice. Following administration of the ASBTi, the fecal bile acid species distribution became remarkably similar in the CSAA and CDAA diet-fed mice (Figure 2A). Increases were observed in excretion of the primary bile acid αMCA and secondary bile acids DCA, lithocholic acid (LCA), and hyodeoxycholic acid (HCA). The differences in fecal excretion of βMCA and ωMCA between the CSAA and CDAA-fed mice were lost after ASBTi treatment (Figures 2B,C). In addition, DCA becomes the major fecal bile acid, likely reflecting increases in bacterial 7α-dehydroxylation and possibly decreased hepatic rehydroxylation of DCA to CA by the enzyme Cyp2a12 (30), and this was not affected by feeding a choline-deficient diet.

**Figure 3:**
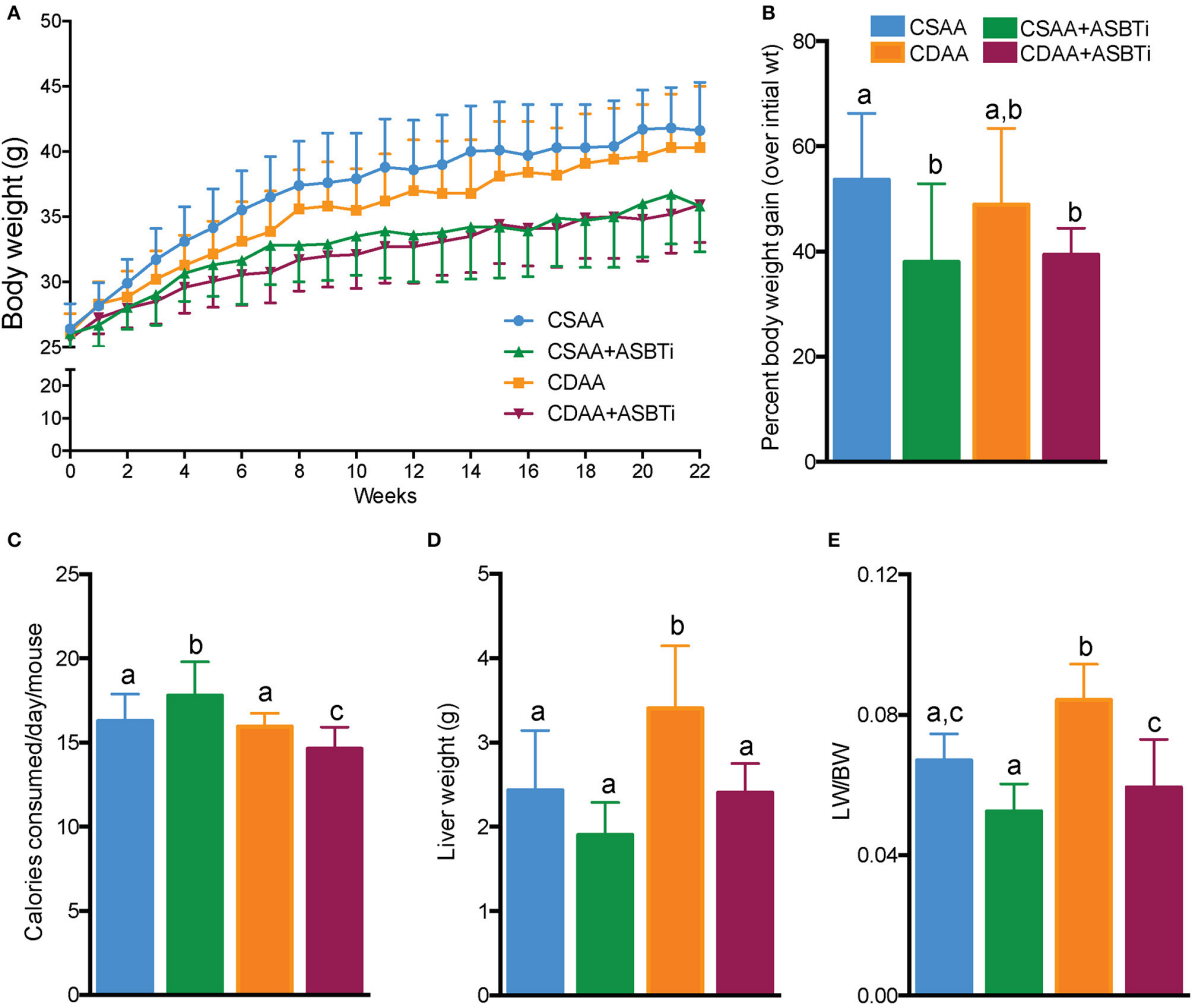
ASBTi treatment decreases bodyweight gain in CSAA and CDAA diet-fed mice and reduces liver weight in CDAA diet-fed mice. **(A)** Bodyweight gain over time in *ad libitum* fed mice, **(B)** Bodyweight gain as percentage of initial bodyweight after 22 weeks, **(C)** Calories consumed per mouse per day, **(D)** Absolute liver weights after 22 weeks, **(E)** Liver to bodyweight ratio; Means ± SD are shown. Distinct lowercase letters indicate significant differences between groups. *P* < 0.05; *n* = 9–12 per group.

To assess whether dietary choline deficiency and/or ASBTi treatment affected body mass, we recorded bodyweight each week (Figure 3A). Bodyweight gain was similar for mice fed the CSAA and the CDAA diets. After 22 weeks, the increase in bodyweight was 54% and 49% (*P* = 0.8, Figure 3B) in the CSAA and CDAA diet-fed mice, respectively. The ASBTi treated groups gained less weight over the course of 22 weeks, irrespective of the diet, significantly reducing bodyweight gain by ~38 and 39% in the CSAA and CDAA diet-fed mice. The caloric intake was slightly higher in the ASBTi-treated CSAA group (17.8 vs. 16.3 kcal/24 h/mouse in the untreated CDAA group, *P* = 0.006) and slightly lower in the ASBTi-treated CDAA group (14.6 vs. 16 kcal/24 h/mouse in the untreated CDAA group, *P* = 0.013, Figure 3C). Hepatomegaly due to steatosis and inflammation is a common feature of NAFLD. After 22 weeks, both absolute liver weight (2.4 vs. 3.4 g, *P* = 0.001, Figure 3D) and liver to bodyweight ratio (0.06 vs. 0.08, *P* < 0.001, Figure 3E) was increased in mice on the CDAA vs. CSAA diet. On the CSAA diet, there was a trend toward a reduced liver weight (1.9 vs. 2.4 g, *P* = 0.2, Figure 3D) and liver to bodyweight ratio (0.052 vs. 0.067, *P* = 0.45) with ASBTi treatment (Figure 3E). On the CDAA diet, liver weight (2.4 vs. 3.4, *P* = 0.001, Figure 3D) and liver to bodyweight ratio (0.059 vs. 0.084, *P* = 0.001, Figure 3E) were significantly lower with ASBTi treatment, and decreased to values similar to that in CSAA diet-fed mice.

### ASBT Inhibitor Treatment Reduces Hepatic Steatosis in CSAA but Not CDAA Diet-Fed Mice

**Figure 4:**
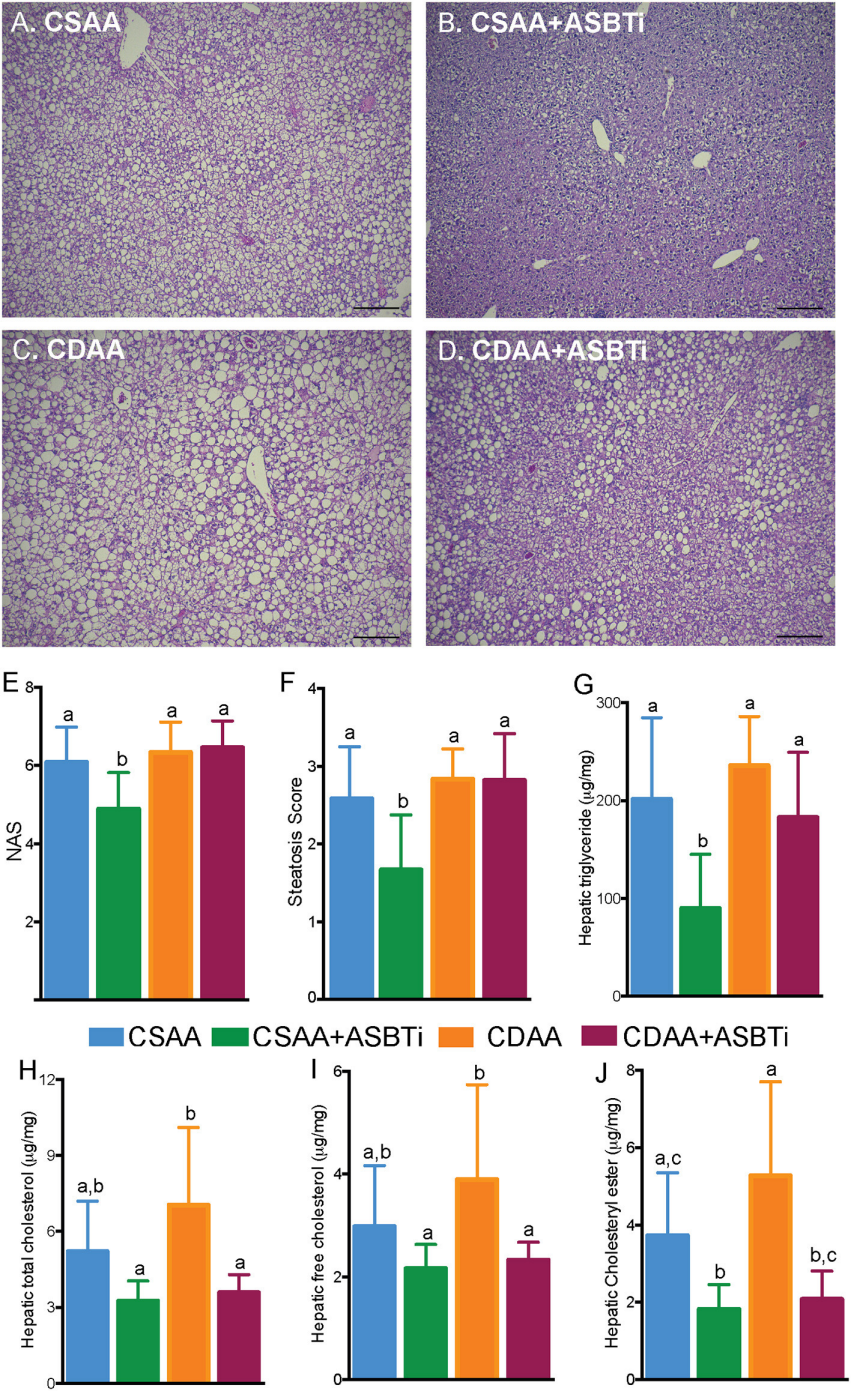
ASBTi treatment reduces hepatic steatosis in CSAA but not CDAA diet-fed mice. Hemotoxylin and eosin stained liver sections from **(A)** CSAA, **(B)** CSAA+ASBTi, **(C)** CDAA, and **(D)** CDAA+ASBTi fed mice after 22 weeks; Scale bars = 100 μm, representative samples; **(E)** Non-alcoholic fatty liver disease Activity Score (NAS), **(F)** Steatosis score, **(G)** Hepatic triglyceride content, **(H)** Hepatic total cholesterol content, **(I)** Hepatic free cholesterol content, **(J)** Hepatic cholesteryl ester content. Means ± SD are shown. Distinct lowercase letters indicate significant differences between groups. *P* < 0.05; *n* = 9–12 per group.

To assess key features of the pathophysiology and progression of NAFLD, we examined the liver histology of the CSAA and CDAA diet-fed mice. Untreated mice fed the CSAA or CDAA diet showed clear features of NAFLD, including steatosis, lobular inflammation and hepatocyte ballooning (Figures 4A,C). Treatment with the ASBTi reduced visible lipid accumulation in mice fed the CSAA diet (Figure 4B) whereas the effects on lipid accumulation were attenuated in mice fed the CDAA diet (Figure 4D). Slides were assessed by a certified veterinary pathologist (author S.G.), who was blinded to the 4 groups, to determine NAFLD activity (NAS) and steatosis scores (Figures 4E,F). NAFLD activity (6.1 vs. 6.3, *P* = 0.9, Figure 4E) and steatosis scores (2.6 vs. 2.8, *P* = 0.7, Figure 4F) were similar for mice fed the CSAA and CDAA diets. ASBTi treatment significantly reduced the NAS and steatosis in mice fed the CSAA but not CDAA diet. To quantify hepatic lipid accumulation, we measured hepatic triglyceride and cholesterol content biochemically. In agreement with the NAS assessment, hepatic triglyceride levels were similar in mice fed the CSAA and CDAA diets (201 vs. 236 μg/mg liver wet weight, *P* = 0.6, Figure 4G). ASBTi treatment significantly reduced hepatic triglyceride accumulation for mice fed the CSAA diet (90 vs. 201 μg/mg liver wet weight, *P* = 0.002, Figure 4G), but the ASBTi-associated reduction in hepatic triglyceride was attenuated in mice fed the CDAA diet (183 vs. 236 μg/mg liver wet weight, *P* = 0.2, Figure 4G). Total hepatic cholesterol content in the liver was similar between the mice fed the low cholesterol-containing CSAA and CDAA diets (5.2 vs. 7.0 μg/mg liver wet weight, *P* = 0.1, Figure 4H). Upon ASBTi treatment, hepatic total (3.6 vs. 7.0 μg/mg liver wet weight, *P* < 0.001) and free cholesterol (2.3 vs. 3.9 μg/mg liver wet weight, *P* = 0.01) levels in the CDAA diet-fed mice were reduced and a similar trend was observed for the CSAA diet (Figures 4H,I). Cholesteryl esters were significantly reduced upon ASBTi treatment for both CSAA and CDAA-fed mice (Figure 4J). Altogether, these show that ASBTi treatment reduces hepatic steatosis on the CSAA diet, in agreement with previous observations (10, 11). However, choline deficiency in the CDAA diet attenuated these effects.

### ASBT Inhibitor Treatment Worsens Hepatic Fibrosis in CDAA Diet-Fed Mice

**Figure 5:**
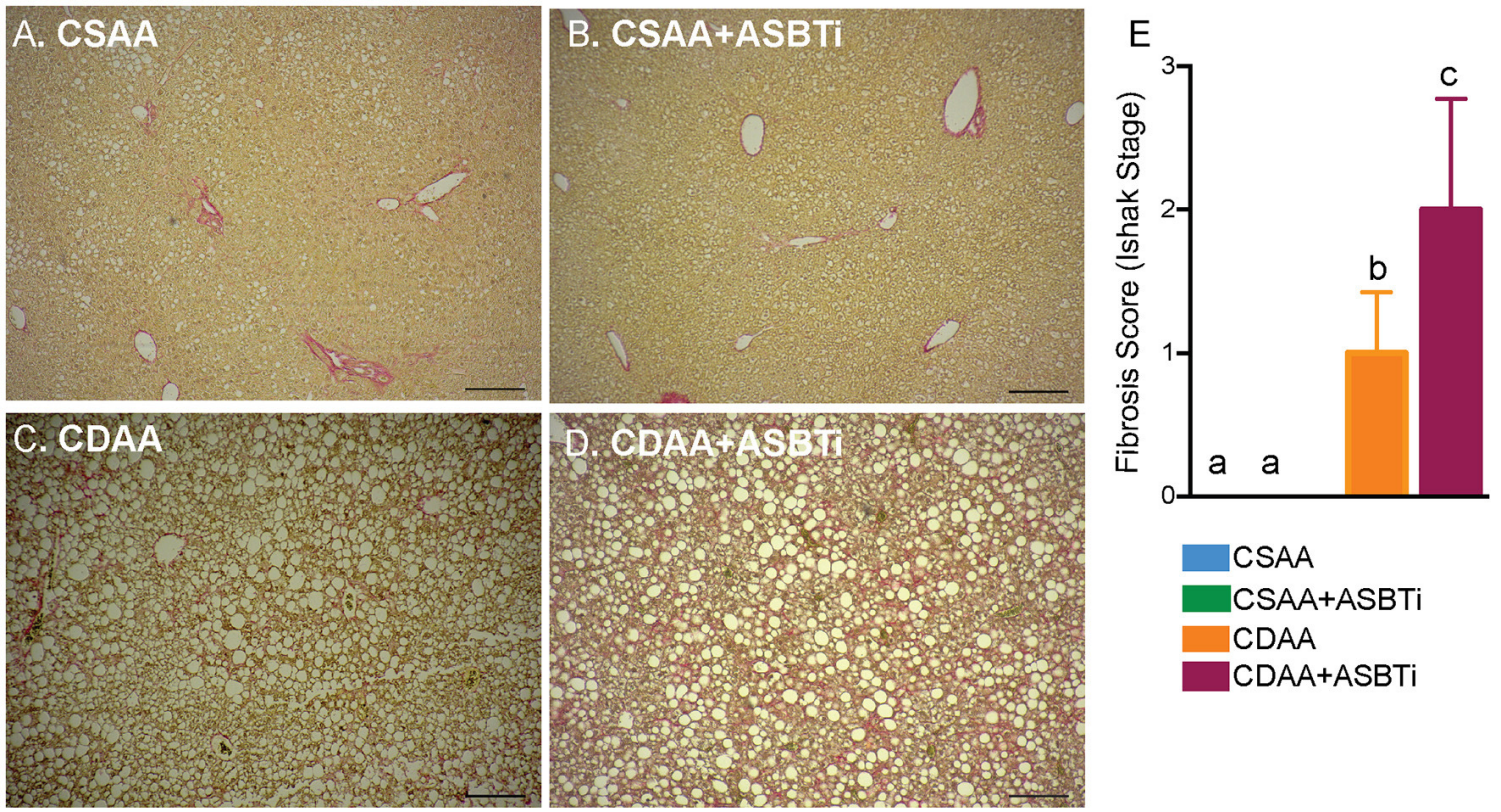
ASBTi treatment increases hepatic fibrosis in CDAA diet-fed mice. Sirius red stained liver sections from **(A)** CSAA, **(B)** CSAA+ASBTi, **(C)** CDAA, and **(D)** CDAA+ASBTi fed mice after 22 weeks; Scale bars=100 μm, representative samples; **(E)** Fibrosis score (Ishak stage). Means ± SD are shown. Distinct lowercase letters indicate significant differences between groups. *P* < 0.05; *n* = 9–12 per group.

The CDAA diet has been shown to successfully induce fibrosis after feeding for 22 weeks (18, 19, 31). To visualize fibrosis on histology, collagen was stained with Sirius Red. The CSAA diet did not induce visually evident fibrosis, and treatment with the ASBTi had no visual impact on histologic Sirius Red staining (Figures 5A,B). For the CDAA diet, hepatic fibrosis was readily apparent irrespective of ASBTi treatment (Figures 5C,D). To quantify the degree of fibrosis, slides were analyzed and scored by a certified veterinary pathologist (author S.G., blinded to diet and treatment groups) using the Ishak Scoring system (24, 32). Livers of mice on the CSAA diet with or without ASBTi did not show any signs of fibrosis and received an Ishak score of 0. Mice on the CDAA diet had significantly higher fibrosis compared to the CSAA diet and received an average Ishak score of 1 (Figure 5E, *P* < 0.001), which corresponds to a Sirius Red stained proportion of 3.0% and fibrous expansion of *some* of the portal areas with or without fibrous septa. ASBTi treatment of the CDAA diet-fed mice resulted in a significantly higher Ishak score of 2 (Figure 5E, *P* = 0.02), which corresponds to a Sirius Red stained proportion of 3.6% and fibrous expansion to *most* of the portal areas with or without short fibrous septa.

**Figure 6:**
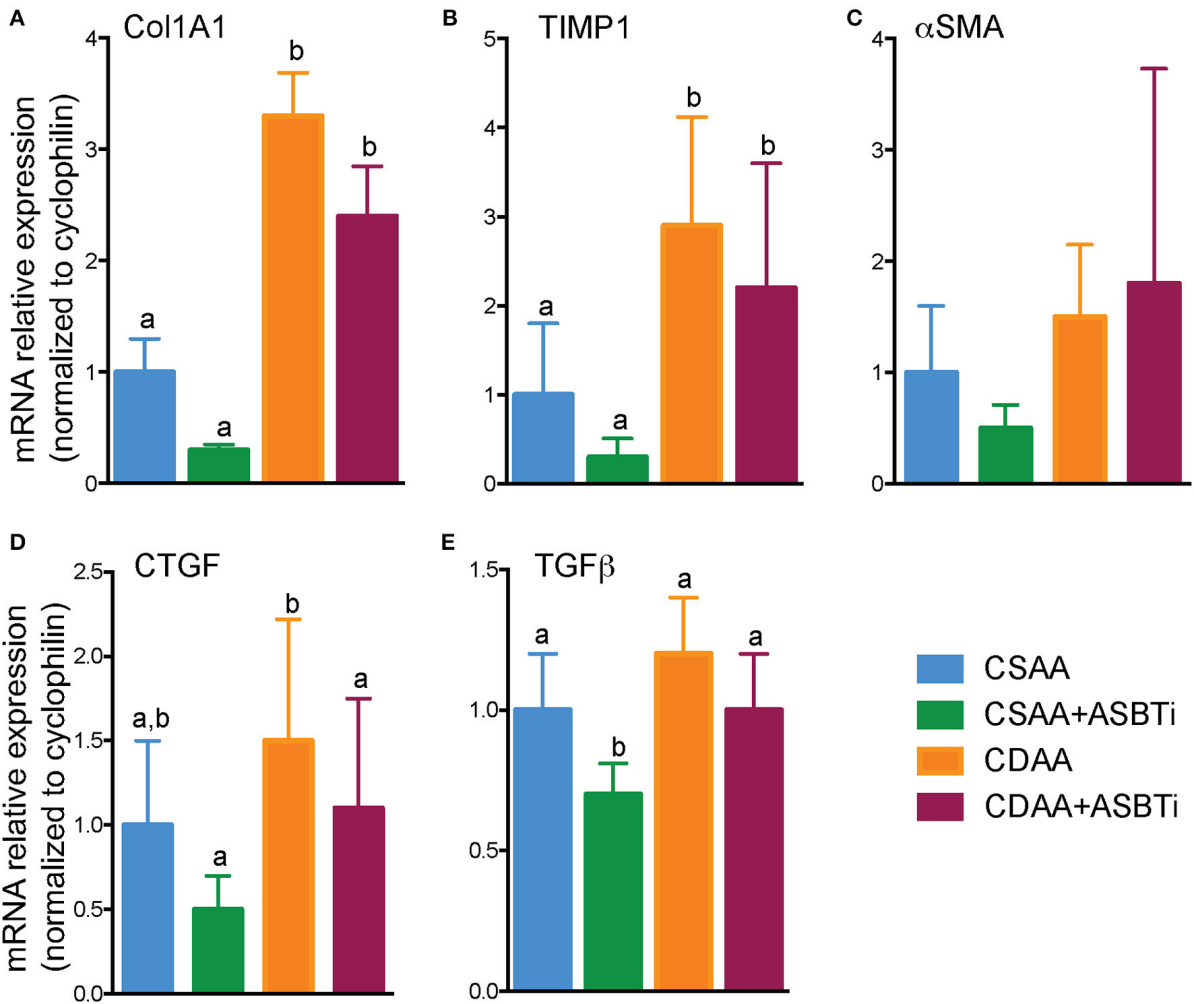
Effect of ASBTi treatment on hepatic gene expression related to fibrosis in mice fed a CSAA and CDAA diet. **(A–E)** Relative hepatic mRNA expression of genes related to fibrosis, normalized to cyclophilin. Distinct lowercase letters indicate significant differences between groups. Means ± SD are shown. *P* < 0.05; *n* = 9–12 per group.

Hepatic mRNA expression of a panel of fibrosis related genes, shown previously to be upregulated by feeding the CDAA diet (31), was measured to assess whether the histological changes were reflected by the gene expression. Collagen type I alpha 1 (*Col1a1*), encoding the main component of collagen type 1 fibers, was upregulated in the CDAA diet and unaffected by ASBTi treatment (Figure 6A). Hepatic mRNA expression of tissue inhibitor of metalloproteinase-1 (*TIMP-1*), a protein involved in degradation of extracellular matrix and promoting proliferation, was similarly increased in the mice fed the CDAA diet and CDAA diet plus the ASBTi (Figure 6B). Interestingly, alpha-smooth muscle actin (α*-SMA*) mRNA, a marker used for hepatic stellate cell activation (33), was unaffected by diet or treatment (Figure 6C). Connective tissue growth factor (CTGF) and transforming growth factor beta (TGFβ) play important roles in fibrogenesis. Hepatic *CTGF* mRNA showed a trend toward increased expression in the CDAA vs. CSAA diet-fed mice, and was significantly decreased with ASBTi treatment in the CDAA diet-fed mice (Figure 6D). *TGF*β was similar between the CSAA and CDAA diets (Figure 6E). ASBTi treatment significantly lowered hepatic *TGF*β expression for the CSAA but not on the CDAA diet-fed mice.

**Figure 7:**
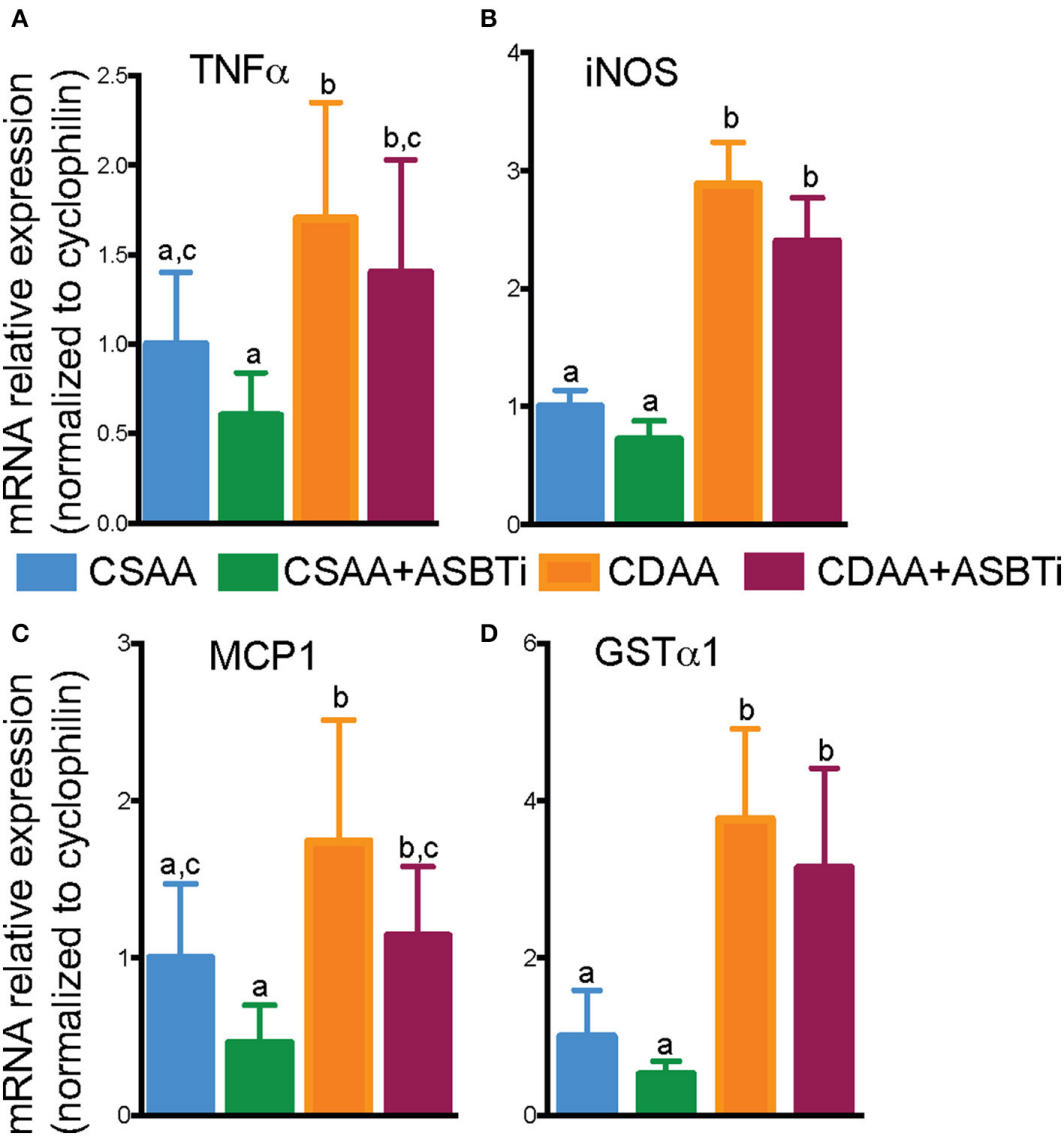
Effect of ASBTi treatment on hepatic gene expression related to inflammation in mice fed a CSAA and CDAA diet. **(A–D)** Relative hepatic mRNA expression of genes related to inflammation, normalized to cyclophilin. Means ± SD are shown. Distinct lowercase letters indicate significant differences between groups. *P* < 0.05; *n* = 9–12 per group.

As progression of liver steatosis to fibrosis generally involves inflammation, we measured hepatic gene expression of several important inflammatory genes. Expression of the proinflammatory genes tumor necrosis factor α (*TNF*α), inducible nitric oxide synthase (*iNOS*), and monocyte chemoattractant protein 1 (*MCP1*) were upregulated in the CDAA vs. CSAA diet-fed mice and unaffected by ASBTi treatment (Figures 7A–C). Hepatic cell damage and increases in inflammation are often paired with oxidative stress. Therefore, we measured glutathione S-transferase A1 (*GST*α*1*) expression, the gene encoding a key enzyme in the anti-oxidative glutathione pathway. *GST*α*1* expression was increased upon feeding the CDAA vs. CSAA diet but unaffected by ASBTi treatment (Figure 7D). Altogether, these data suggest that the ASBTi treatment was not able to prevent inflammation, oxidative stress or fibrosis in this dietary choline deficiency mouse model of NAFLD/NASH.

### ASBT Inhibitor Treatment Reduces Intestinal Fat Absorption in CSAA but Not CDAA Diet-Fed Mice

**Figure 8:**
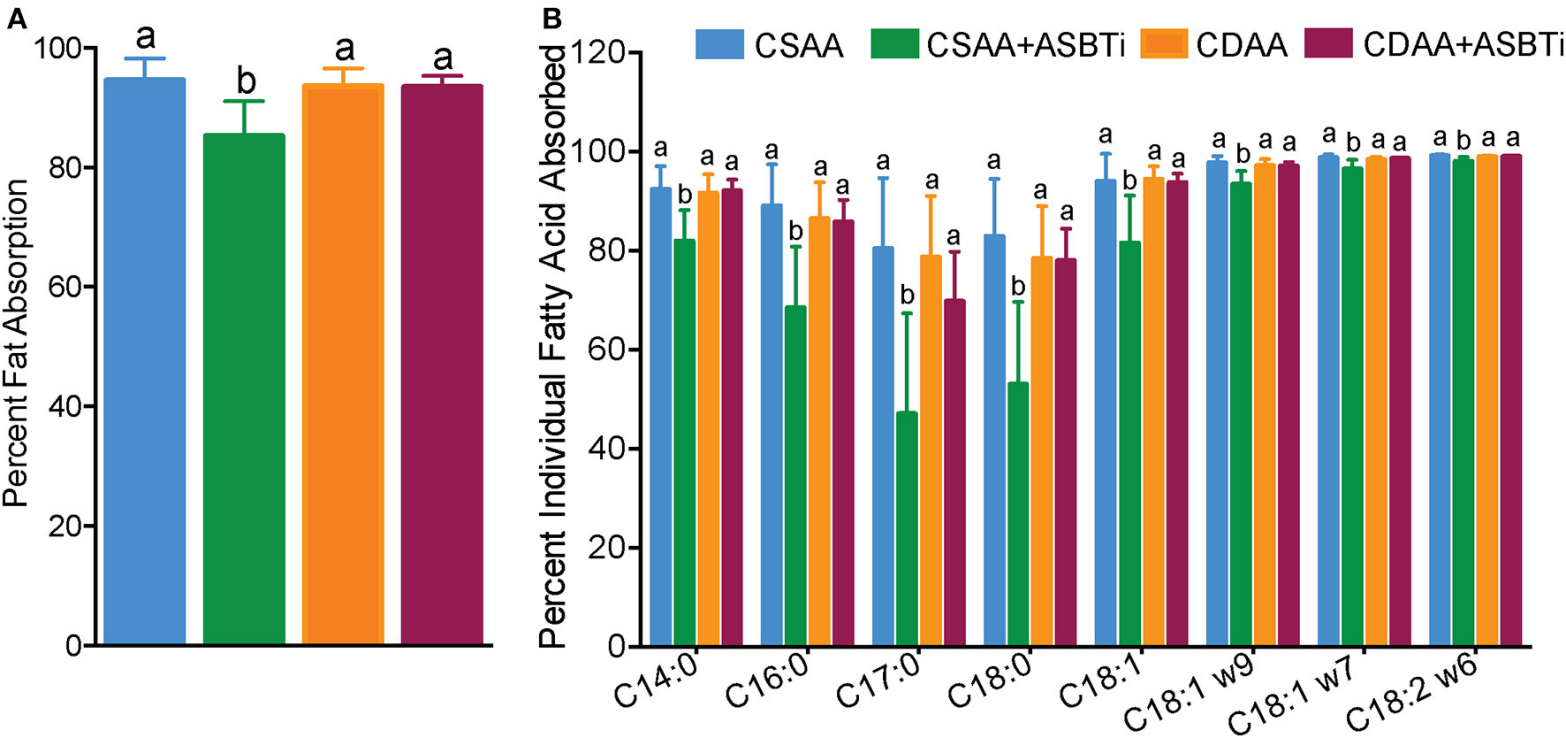
ASBTi treatment reduces intestinal fat absorption in mice fed a CSAA but not CDAA diet. **(A)** Percentage of total intestinal fat absorption, **(B)** Percentage of intestinal absorption of individual fatty acid. Distinct lowercase letters indicate significant differences between groups. Distinct lowercase letters indicate significant differences between groups. *P* < 0.05; *n* = 9–12 per group.

**Figure 9:**
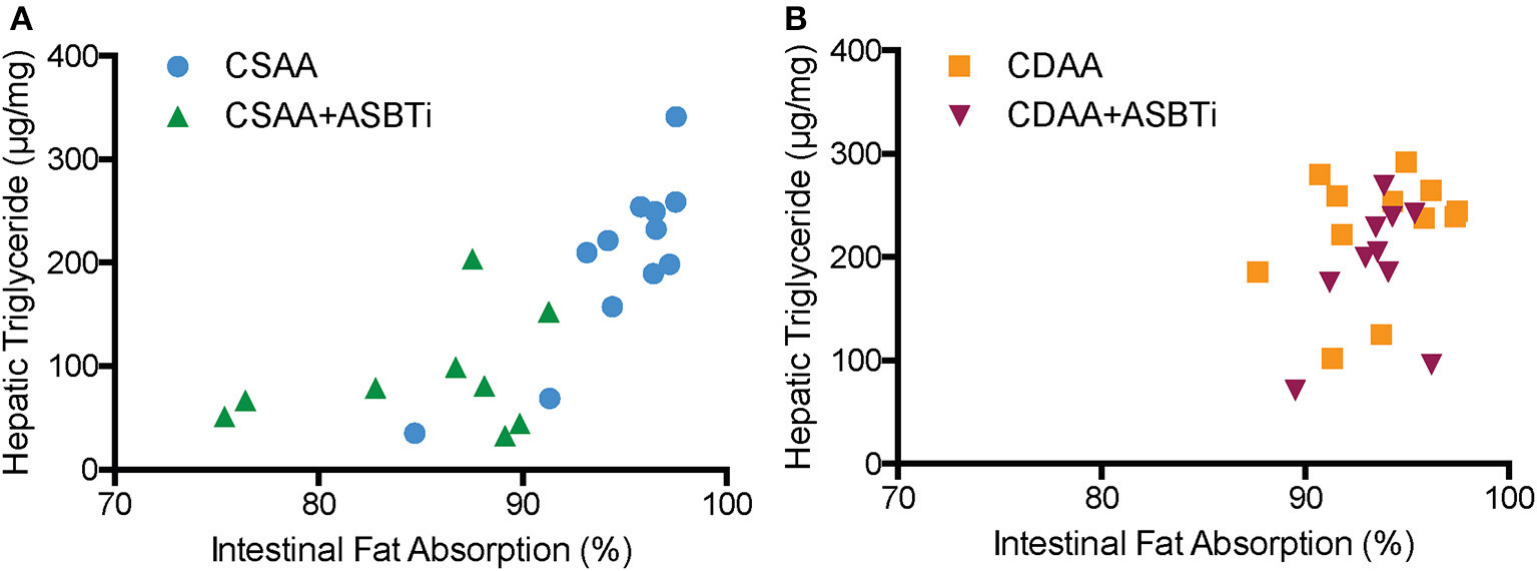
Correlation of hepatic triglyceride content to intestinal fat absorption. **(A)** Hepatic triglyceride content correlated to intestinal fatty acid absorption in CSAA and CSAA+ASBTi fed mice (Spearman *R* = 0.75, *P* < 0.0001) **(B)** Hepatic triglyceride content did not correlate to intestinal fatty acid absorption in CDAA and CDAA+ASBTi fed mice (Spearman *R* = 0.32, *P* = 0.13 *n* = 9–12 per group).

We previously reported that genetic ASBT knockout in mice reduces intestinal fat and cholesterol absorption (29, 34, 35). In order to determine if the ASBTi treatment had similar effects in the CSAA and CDAA diet-fed mice, we measured fat absorption using the sucrose polybehenate method (20). For mice on the CSAA diet, ASBTi treatment reduced total fatty acid absorption by almost 10% (85.3 vs. 94.6%, *P* < 0.001, Figure 8A). The ASBTi treatment-associated decrease in fat absorption in the CSAA diet-fed mice was greater for the saturated fatty acids myristic acid (C14:0), palmitic acid (C16:0), margaric acid (C17:0), and stearic acid (C18:0) followed by the *trans* saturated fatty acid, elaidic acid (C18:1 ω9) (Figure 8B). For the group of saturated fatty acids, the ASBTi treatment effect on absorption increased with fatty acid acyl chain length, which corresponds to greater hydrophobicity and correlates with increased bile acid requirement for efficient micellar solubilization (36). The ASBTi treatment-associated decreases in fat absorption in the CSAA diet-fed mice for the mono- and polyunsaturated fatty acids oleic acid (C18:1ω9), vaccenic acid (C18:1ω7), and linoleic acid (C18:2ω6) were also statistically significant but the absolute differences were smaller (Figure 8B). We found no difference in total fat absorption between the mice fed the CSAA and the CDAA diets (94.6 vs. 93.6%, *P* = 0.9, Figure 8A). However, in contrast to mice fed the CSAA diet, absorption of neither total fat (93.5 vs. 93.6%, *P* = 1.0, Figure 8A) or the individual fatty acids were affected by ASBTi treatment of CDAA diet-fed mice. Correlation analysis showed that the hepatic triglyceride content is highly correlated to intestinal fatty acid absorption in mice fed the CSAA (Spearman *R* = 0.75, *P* < 0.001, Figure 9A) but not CDAA diet (Spearman *R* = 0.32, *P* = 0.9, Figure 9B).

Although we did not directly assess cholesterol absorption in the current study, we measured fecal excretion of neutral sterols, cholesterol and its fecal metabolites. Fecal neutral excretion (μmol/24 h/100 g BW, mean ± SD, *n* = 9–12 per group) was significantly higher in CSAA mice treated with an ASBTi compared to CSAA control mice (CSAA plus ASBTi: 12.0 ± 4.8 vs. CSAA: 7.5 ± 3.3, *P* = 0.02). On the CDAA diet, fecal neutral sterol excretion was comparable to the CSAA diet (CDAA: 9.7 ± 3.2 vs. CSAA:7.5 ± 3.3, *P* = 0.4**)**. However, in contrast to the CSAA diet, ASBTi treatment did not significantly increase fecal neutral sterol excretion in mice fed the CDAA diet (CDAA plus ASBTi: 8.0 ± 1.2 vs. 9.7 ± 3.2, *P* = 0.6). Previous studies have suggested that feeding a choline-deficient diet can promote triglyceride accumulation in enterocytes as a result of impaired chylomicron secretion (37, 38). In this study, there was a trend toward increased jejunal tissue triglyceride in CDAA vs. CSAA diet-fed mice, but the differences were not statistically significant (*P*> 0.05) and there was no effect of ASBTi treatment on the jejunal tissue triglyceride (μg per g tissue, mean ± SD, *n* = 9–12 per group; CSAA: 23.2 ± 10.5; CSAA plus ASBTi: 22.8 ± 9.2; CDAA: 29.8 ± 9.2; CDAA plus ASBTi: 33.01 ± 10.7). The jejunal tissue total cholesterol content (μg per g tissue, mean ± SD, *n* = 9–12 per group) was also not different (*P* > 0.05) between the different groups (CSAA: 2.5 ± 0.2; CSAA plus ASBTi: 2.31 ± 0.23; CDAA: 2.27 ± 0.28; CDAA plus ASBTi: 2.22 ± 0.39).

## Discussion

In the current study, we showed that treatment with an ASBTi did not prevent development of hepatic fibrosis in a CDAA diet-fed mouse model. Additionally, under dietary choline-deficient conditions, previously reported effects of ASBT inhibition on fat and cholesterol absorption were nullified, possibly contributing to the phenotype. Dietary interventions in mouse models to reproducibly induce NASH and fibrosis are limited. The CDAA diet was used in the current study because, contrary to the MCD diet, the CDAA diet was shown to successfully induce hepatic fibrosis without significantly lowering bodyweight and plasma glucose levels (18, 19). Approximately 95% of the choline in animal tissues is present as phosphatidylcholine (PC), a major component of membranes (39). The primary mechanism believed to be involved in the effects of choline deficiency on hepatic steatosis is impairment of VLDL secretion due to insufficient PC (15). Interestingly, bile acid sequestrants act similar to ASBT inhibition by interrupting the enterohepatic circulation of bile acids, and have been shown to increase plasma VLDL and triglyceride levels (40–42). Therefore, one of the mechanisms contributing to the anti-steatotic actions of ASBTi treatment may be increasing hepatic VLDL secretion and triglyceride export from the liver, which is attenuated under choline-deficient conditions.

In addition to affecting VLDL secretion, choline deficiency alters mitochondrial function (43), fatty acid beta-oxidation (44), and changes epigenetic marking (45). Choline deficiency also alters the intestinal microbiota, which may be involved in the development of NAFLD (46). Treatment with the ASBTi induces similar changes in the fecal bile acid profile for the CSAA and CDAA diet-fed mice, such that the fecal bile acid composition including the microbiota-derived secondary bile acids is almost identical for the two ASBTi-treated groups. Changes in the gut microbiota composition associated with feeding the choline-deficient amino acid-defined diet have been reported previously (47). However, the almost identical fecal secondary bile acid profiles suggests that at least this aspect (bile acid biotransformation) of the gut microbiome actions does not appear to account for the differential response of the CSAA and CDAA diet-fed mice to the ASBTi. It was previously shown that treatment of high fat diet-fed mice with the ASBTi for 16 weeks shifted the hepatic bile acid composition shifted toward a more hydrophobic and thus potentially cytotoxic profile (10). *In vitro* studies have shown that PC protects hepatocytes from bile acid-induced cytotoxicity (48, 49). Although not injurious when hepatic PC synthesis is intact, the ASBTi-induced shift to a more hydrophobic bile acid pool may be injurious under PC-deficient conditions. The absence of beneficial effects in the CDAA model has been noted for other therapeutic interventions such as FXR agonism with obeticholic acid (50). Moreover, feeding a choline-deficient, iron-supplemented L-amino acid-defined diet to rats has been shown to induce a persistent fibrosis, potentially secondary to increased oxidative stress (51). This later observation is particularly important since it has recently been shown that interrupting the enterohepatic circulation of bile acids in mice decreases hepatic glutathione levels and impairs glutathione regenerating capacity (52). This is due in part to increased expression of cysteine dioxygenase type 1, in order to shunt cysteine toward taurine biosynthesis to meet the increased demand for synthesis of taurine-conjugated bile acids. A decrease in hepatic cysteine and glutathione levels combined with choline deficiency may underlie the increase in fibrosis observed in the ASBTi-treated CDAA diet-fed mice. However, these results should be interpreted with caution in regard to human NAFLD/NASH pathophysiology, as this rarely involves choline deficiency (53) and humans preferentially use glycine vs. taurine for bile acid conjugation (54). Future studies using different models are needed for understand the potential impact of ASBTi treatment on NASH and fibrosis. Fortunately, several recently developed murine NAFLD models have shown promising results in mimicking human NAFLD/NASH pathophysiology (55, 56).

Anti-steatotic effects of ASBT pharmacological inhibition have been a consistent finding in high fat diet-fed mouse models, including the CSAA diet-fed mice in this study (10, 11), and we previously observed inhibitory effects of ASBT genetic inactivation on intestinal cholesterol and fat absorption (29, 34, 35). In our current study, ASBTi treatment lowered fatty acid absorption and intestinal fat absorption positively correlated with hepatic triglyceride levels in the CSAA diet-fed mice. By contrast in the choline-deficient CDAA diet-fed mice, ASBTi-treatment had no effect on intestinal fat absorption and the hepatic anti-steatotic effects were attenuated. The mechanisms underlying our observations in the CDAA-fed mice remain unclear. Intestinal intraluminal PC concentrations contribute to mixed micelle formation, lipid transport across the unstirred water layer, and subsequent lipid translocation to the brush border membrane (57). In rats, dietary choline deficiency was shown to decrease chylomicron secretion and alter intestinal cell morphology and physiology, resulting in impairments of dietary fat absorption (37, 38). One could speculate that intestinal adaptations present in the choline-deficient CDAA diet-fed mice to compensate for decreased biliary PC affect lipolytic and post-lipolytic events related to intestinal lipid absorption. The absence of a protective effect on hepatic steatosis for interventions that act via reduced intestinal fat absorption has been observed previously in dietary choline-deficient models. For example, intestine-specific knockout of the zinc finger transcription factor GATA4 reduces intestinal lipid absorption and was protective against hepatic steatosis induced by feeding a high fat but not MCD diet (58).

In conclusion, this study showed that ASBTi treatment did not affect intestinal fat absorption and was not protective against hepatic fibrosis in a choline-deficient mouse model of NASH. Nevertheless, these findings add to our understanding of the uses and limitations of dietary choline-deficient mouse and the potential mechanisms by which interrupting the enterohepatic circulation of bile acids may impact development of NAFLD and NASH. Indirectly modulating intestinal fat absorption via ASBTi treatment is an interesting potential therapeutic target. Future studies using different models for NASH are warranted to further understand the relationship between bile acids and the progression of hepatic steatosis to NASH and fibrosis.

## Data Availability Statement

The datasets generated for this study are available on request to the corresponding author.

## Ethics Statement

The animal study was reviewed and approved by Emory University Institutional Animal Care and Use Committee.

## Author Contributions

AR, IP, and SG performed the experiments and collected and analyzed the data. AR managed the study. AR, SK, and PD designed the study and conceived the experiments. PD, IP, AR, and SK wrote the paper.

### Conflict of Interest

The authors declare that the research was conducted in the absence of any commercial or financial relationships that could be construed as a potential conflict of interest.

## Abbreviations

ASBT: Apical sodium-dependent bile acid transporter
ASBTi: Apical sodium-dependent bile acid transporter inhibitor
BW: bodyweight
CA: cholic acid
CDAA: choline-deficient, L-amino acid-defined
CSAA: choline-sufficient L-amino acid-defined
DCA: deoxycholic acid
FXR: farnesoid-X receptor
GC: gas chromatography
HCA: hyodeoxycholic acid
IBAT: ileal bile acid transporter
LCA: lithocholic acid
MCA: muricholic acid
MCD: methionine/choline-deficient
NAFLD: non-alcoholic fatty liver disease
NAS: NAFLD Activity Score
NASH: non-alcoholic steatohepatitis
PC: phosphatidylcholine
VLDL: very low density lipoprotein.
